# Recovery of a hypolipidemic polysaccharide from artificially cultivated *Sanghuangporus vaninii* with an effective method

**DOI:** 10.3389/fnut.2022.1095556

**Published:** 2023-01-13

**Authors:** Zuo-fa Zhang, Ting-ting Song, Jian-fei Chen, Guo-ying Lv

**Affiliations:** Institute of Horticulture, Zhejiang Academy of Agricultural Science, Hangzhou, China

**Keywords:** *Sanghuangporus vaninii*, hypolipidemic polysaccharide, acid-ethanol pretreatment, zebrafish model, structure illustration

## Abstract

In this study, an effective method was developed to extract the polysaccharide from *Sanghuangporus vaninii* (PFSV) by destroying the cell wall. Box-Behnken design was employed to determine the optimal processing conditions as follows: processing temperature (80°C), processing time (0.81 h) and amount of HCl (1.5 ml). Under these conditions, the yield of PFSV reached 5.94 ± 0.16%. The purified polysaccharide (PFSV-2) was found to be a hetero-polysaccharide with an average molecular weight of 20.377 kDa. The backbone of PFSV-2 was composed of an →6)-*α*-Gal*p*-(1→ and →2,6)-*β*-Man*p*-(1→ and →2)-*α*-Fuc*p*-(1→ and was branched of t-*α*-Man*p*-(1→ at position 2 of residue B. PFSV-2 showed hypolipidemic activity by decreasing lipid accumulation and the levels of total cholesterol and triglycerides in zebrafish larvae. Furthermore, PFSV-2 downregulated the *pparg, fasn*, and *HMGCRb* genes and upregulated the *pparab* and *acaca* genes. These findings suggested PFSV-2 may be a promising candidate in lipid regulation therapy.

## Introduction

Hyperlipidemia, a lipid metabolic disorder, is considered to be the major risk factor for fatty liver, hypertension, myocardial infarction, atherosclerosis, stroke and cerebrovascular diseases (1).

Reactive oxygen species (ROS), oxidative stress, and inflammation are the main reasons for hyperlipidemia. ROS can cause oxidative stress and lipid peroxidation, which are involved in the cellular damage to produce dyslipidemia and related diseases (2). In addition, the overexpression of tumor necrosis factor, interleukins-6 and C-reactive protein induced by inflammations can cause the elevation of the lipids and cholesterols level in the circulation (3). Therefore, it is important to find a way to prevent the hyperlipidemia. To date, some synthetic hypolipidemic drugs have been used to treat hyperlipidemia, however, their negative side effects hamper their applications. Increasing attention has therefore been focused on the discovery of natural alternative with good hypolipidemic properties.

Natural polysaccharides are widely distributed in plants, animals and microbes. They have attracted research interest due to their unique nutritional value and various health promotion functions (4, 5). Mushrooms and their extracts have long been used in folk medicine due to their widely nutraceutical and medicinal properties. *Sanghuangporus vaninii* is a well-known wood rot fungus that is widely cultured in China. Polysaccharides are the main bioactive components in *S. vaninii* (PFSV) and they exhibit various biological functions (6). In our preliminary study, we found that crude PFSV enjoyed obviously lipid-lowering activity. Studying on the chemical structures and chain conformations of polysaccharides is important to understand the hypolipidemic activity of PFSV. The chemical structures of PFSV are complex, and may be implicated in various hypolipidemic mechanisms. To make more effective use of *S. vaninii* resources, it is necessary to investigate the structure and hypolipidemic activity of polysaccharides from *S. vaninii*.

The efficient extraction of the active polysaccharides is crucial, as a high yield of polysaccharide is desirable for the development of new polysaccharide-based applications and products. However, the close association with cellulose and lignin through physical and chemical bonds makes it challenging to extract the polysaccharide from *S. vaninii*. Therefore, it is important to destroy the cell wall to enable the solvent to penetrate the cell wall and enhance the extraction efficiency. Acid hydrolysis may provide an efficient way to break down the cells walls. It has been reported that pretreatment with acid in alcoholic medium at relatively low temperature is an effective and controllable way to destroy the cell wall and change the dissolution mechanisms of polysaccharides (7). Therefore, it was hypothesized that pretreatment with acid in alcoholic medium would be an effective way to extract the polysaccharide from *S. vaninii* and that the purified PFSV would possess pronounced hypolipidemic properties.

In the present study, a new Box-Behnken design (BBD) for optimizing the acid pretreatment of the fungal cell wall in alcohol was developed. The PFSV was extracted and purified, structural analysis was performed and the lipid-lowering activity of the purified polysaccharide and the underpinning mechanism were also investigated.

## Materials and methods

### Materials and reagents

The fruiting body of *S. vaninii* was harvested from Zhejiang Academy of Agricultural Science farm after it was grown for 4 months. The mushroom was dried at 60°C in an oven, pulverized and passed through a 60-mesh screen for subsequent use. Dextrans (Dextran T1000, T500, T70, T40, and T10) were purchased from the American Polymer Standards Corp (Mentor, OH, USA). Monosaccharide standards (arabinose, fucose, mannose, rhamnose, glucose, galactose, xylose, galacturonic acid, and glucuronic acid), Coomassie Brilliant Blue G-250, trifluoroacetic acid and simvastatin were obtained from Sigma-Aldrich (St. Louis, MO, USA). DEAE cellulose-52 and Sephacryl S-400HR were supplied by Baoman Biotechnology (Shanghai, China). All chemicals and solvents were reagent grade or better.

### Preparation of crude polysaccharide from *S. vaninii*

Ground *S. vaninii* powder (1.0 g) was heated at reflux in a round-bottom flask with preheated ethanol (92.5 wt%) and hydrochloric acid (37%) in different ratios (8.5 ml:1.5 ml, 9.0 ml:1 ml, and 9.5 ml: 0.5 ml) for various durations (0.5 h, 1 h, and 1.5 h) at different temperatures (60, 70, and 80°C). The mixture was cooled down in an ice-water bath and neutralized with sodium bicarbonate (15%, w/v) following the treatment. The treated mushrooms were separated by centrifugation at 6,000 rpm for 15 min. Subsequent procedures were similar to those for our reported method (8). The yield of polysaccharide was calculated according to the following equation:


$$\begin{array}{l} \text{PFSV yield }\left(\text{\%}\right)=\left(\text{dry PFSV weight}/\text{raw material weight}\right)\times 100 \end{array}$$

BBD was employed to find the optimum conditions based on the preliminary range of extraction variables. Processing temperature (A), processing time (B) and the amount of HCl (C) were chosen to investigate their combined effect. The range and levels of independent variables are presented in Table 1A. Design Expert software (8.0.6) was used for the experimental design, and the variance and regression coefficients of individual linear, quadratic and interaction terms. Seventeen combinations including five replicates of the center point were included in this design. All trials were performed in triplicate and the average polysaccharide yield was taken as the result.

**Table 1A:** The Box-Behnken design matrix and the results for extraction yield of crude polysaccharides from *Sanghuangporus vaninii*.

**Run numbers**	**(A) Processing temperature (°C)**	**(B) Processing time (h)**	**(C) Amount of HCl (ml)**	**Response (%)**
1	0 (70)	−1 (0.5)	−1 (0.5)	4.43 ± 0.28
2	−1 (60)	0 (1)	1 (1.5)	4.58 ± 0.35
3	−1 (60)	1 (1.5)	0 (1)	4.22 ± 0.31
4	1 (80)	0 (1)	1 (1.5)	5.99 ± 0.19
5	0 (70)	0 (1)	0 (1)	4.97 ± 0.24
6	0 (70)	0 (1)	0 (1)	5.01 ± 0.21
7	−1 (60)	−1 (0.5)	0 (1)	4.07 ± 0.36
8	−1 (60)	0 (1)	−1 (0.5)	4.28 ± 0.19
9	0 (70)	1 (1.5)	1 (1.5)	5.1 ± 0.09
10	0 (70)	1 (1.5)	−1 (0.5)	4.81 ± 0.15
11	1 (80)	−1 (0.5)	0 (1)	5.37 ± 0.24
12	0 (70)	0 (1)	0 (1)	4.86 ± 0.26
13	0 (70)	−1 (0.5)	1 (1.5)	5.23 ± 0.19
14	0 (70)	0 (1)	0 (1)	4.78 ± 0.33
15	0 (70)	0 (1)	0(1)	4.94 ± 0.22
16	1 (80)	1 (1.5)	0 (1)	5.20 ± 0.28
17	1 (80)	0(1)	−1 (0.5)	5.81 ± 0.35

Conventional hot water (HWE) and ultrasonic-assisted extraction (UAE) were conducted as control experiments using the previously optimized extraction conditions. For conventional hot water extraction, distilled water (30 ml) was added to pre-treated mushroom powder (1.0 g) and the mixture was heated at reflux with boiling water for 1 h. The supernatant was separated from the solid residue by centrifugation and the above extraction was repeated twice (9). The subsequent procedure was similar to that for the acid-ethanol pretreatment. For ultrasonic-assisted extraction (UAE), distilled water (30 ml) was added to pre-treated mushroom powder (1.0 g) and the mixture was extracted in an ultrasonic cleaner (KQ-500E, Kunshan Ultrasound Instrument Co., Ltd., Jiangsu, China, 40 kHz) at 50°C for 25 min (10). The subsequent procedure was similar to that for the acid-ethanol pretreatment.

### Purification of PFSV

The crude PFSV was first purified through a DEAE-52 cellulose column (2.6 × 60 cm) using H_2_O_2_ and different concentrations of NaCl solutions (0–0.5 M) at 1.0 ml·min^−1^. The fractions were collected in 10-ml aliquots per tube, and the sugar content was determined using the phenol-sulfuric acid assay (11). The second fraction was further purified by a Sephacryl S-400HR column (1.6 × 100 cm) at 0.2 ml·min^−1^ for further investigation (PFSV-2). The main fractions were collected, dialyzed and lyophilized for further use.

### Structural identification of the purified polysaccharide

For FT-IR spectroscopy, PFSV-2 was mixed with KBr and the spectra were recorded from 4,000 to 500 cm^−1^ with a Nicolet 6700 FT-IR spectrometer (Thermo Electron Corp., Waltham, MA, USA).

The monosaccharide composition of PFSV-2 was determined by high-performance anion-exchange chromatography (Dionex LC_30_ equipped with a CarboPac^TM^ PA20 column and a pulsed amperometric detector) (12).

The molecular weight of PFSV-2 was determined by high-performance gel permeation chromatography (HPGPC), using a refractive index detector and a multi-angle laser scattering detector according to the reported method (13).

The methylation of PFSV-2 was performed according to the method of Huang et al. (14). After methylation, derivatives for gas chromatography-mass spectrometric analysis were obtained by hydrolysis, reduction and acetylation of the completely methylated polysaccharide.

The freeze-dried PFSV-2 (50 mg) was dissolved in 500 μl of deuterium oxide (D_2_O). ^1^H, ^13^C, ^1^H-^1^H COSY, NOESY, HSQC and HMBC NMR spectra of PFSV-2 were obtained using a Bruker AV-400 MHz (^13^C) spectrometer, using tetramethylsilane (TMS) as an internal standard according to a previously published method (15).

### Lipid-lowering activity

#### Zebrafish larva treatment

This study was conducted in conformity with the Guide for the Care and Use of Laboratory Animals. Zebrafish larvae were divided into six groups: the control group, egg yolk group, and 3 treated groups at concentration of 0.1, 0.5, and 1.0 mg·ml^−1^ PFSV-2, and the positive group in which simvastatin was used as a comparative agent at 0.025 μg·ml^−1^ (each group had 50 larvae). Fish embryos were generated by natural mating and hatched in fish water at 28°C. To develop a hyperlipidemia model, 5-day post-fertilization zebrafish larvae were fed with 0.1% egg yolk for 48 h. Treatment was performed until 5 dph with drugs administration according to the previously reported method (16). On the final day, the larvae were euthanized and fixed in paraformaldehyde (4%) overnight.

#### Oil red O staining and the triglyceride and total cholesterol levels analysis

The fixed zebrafish larvae washed in phosphate buffer saline (PBS), sequentially infiltrated with 50%, 75%, and 100% 1, 2-propanediol for 10 min, then washed in PBS. Fresh ORO solution (0.5%) was added to dye the larvae for 20 h, after which the larvae were washed with PBS. Stained larvae were stored in 100% glycerol and imaged by using an Olympus SZX 16 microscope.

To determine the triglyceride (TG) and total cholesterol (TC) levels, each group of zebrafish was homogenized with PBS (1:9, w/v) and centrifuged at 4,000 rpm for 10 min at 4°C. The TG and TC levels were then determined according to the manufacturer's instructions (Nanjing Jiancheng, China).

#### RNA extraction and real-time PCR

Total RNA was extracted from 20 fish larvae using an RNA simple Total RNA kit (Tiangen Biotech, China) according to the manufacturer's protocol. The isolated RNA was reversed with HiScript II Q RT SuperMix (Vazyme, China). Real-time quantitative PCR was carried out on a ViiA^TM^ 7 Real-Time PCR system (Applied Biosystems, USA). The primers used for each gene are listed in Supplementary Table S1. PCR reaction was performed following the cycling protocol of 95°C for 15 min, followed by 40 PCR cycles of 95°C for 10 s, 60°C for 20 s and 72°C for 20 s. Dissociation curves were run after amplification to identify the specific PCR products. Light Cycler 480 Software was employed to perform the relative quantification for the expression of target genes.

### Statistical analysis

Data are shown as the mean ± standard deviation (SD). One-way analysis of variance (ANOVA) was used as the statistical analysis technique. The statistical significance of differences between the groups was assessed using the Student's *t*-test.

## Results and discussion

### Optimization of the polysaccharide extraction using RSM

To achieve the highest PFSV yield, BBD constituted 17 experiments for optimization of three individual parameters were performed. The observed and predicted responses of these experiments are presented in Table 1A.

The response variable Y was characterized by the following second-order polynomial equation through analysis of the experimental data with Design-Expert 8.05:


$$\begin{array}{lll} Y & = & 4.91\;+\;0.65A\;+\;0.029B\;+\;0.2C\;-\;0.080AB\;-\;0.03AC\\ & - & \;0.13BC\;+\;0.038A^{2}\;-\;0.23B^{2}\;+\;0.22C^{2} \end{array}$$

Table 1B summarized the results of the analysis of variance, goodness-of-fit and the adequacy of the model. The high *F*-value (22.84) and low *p*-value (0.0002) suggested that the regression model was significant, whereas the lack-of-fit value was insignificant. These results indicated that the model was sufficiently accurate for predicting the variations. The high values of *R*^2^ (0.9671) and $R_{\text{adj}}^{2}$ (0.9647) indicated a high degree of correlation between the experimental and predicted values. The low CV (2.91%) and high value of adequate precision (18.457) suggested that the experiment had a very high degree of precision and reliability (17).

**Table 1B:** ANOVA for response surface quadratic model.

**Variables**	**Sum of squares**	**DF**	**Mean square**	***F***	***p***
Model	4.23	9	0.47	22.84	0.0002
A	3.41	1	3.41	165.62	<0.0001
B	0.0066	1	0.0066	0.32	0.5884
C	0.31	1	0.31	14.98	0.0061
AB	0.026	1	0.026	1.24	0.3014
AC	0.0036	1	0.0036	0.18	0.6882
BC	0.065	1	0.065	3.16	0.1186
A^2^	0.006	1	0.006	0.29	0.6058
B^2^	0.23	1	0.23	11.28	0.0121
C^2^	0.20	1	0.20	9.49	0.0178
Residual	0.14	7	0.021		
*Lack of fit*	0.11	3	0.037	4.33	0.0953
*Pure error*	0.034	4	0.008		
Cor total	4.37	16			
R^2^	0.9671				
Adj R^2^	0.9647				
Pred R^2^	0.5849				
Adeq precision	18.457				
CV%	2.91				

**Figure 1:**
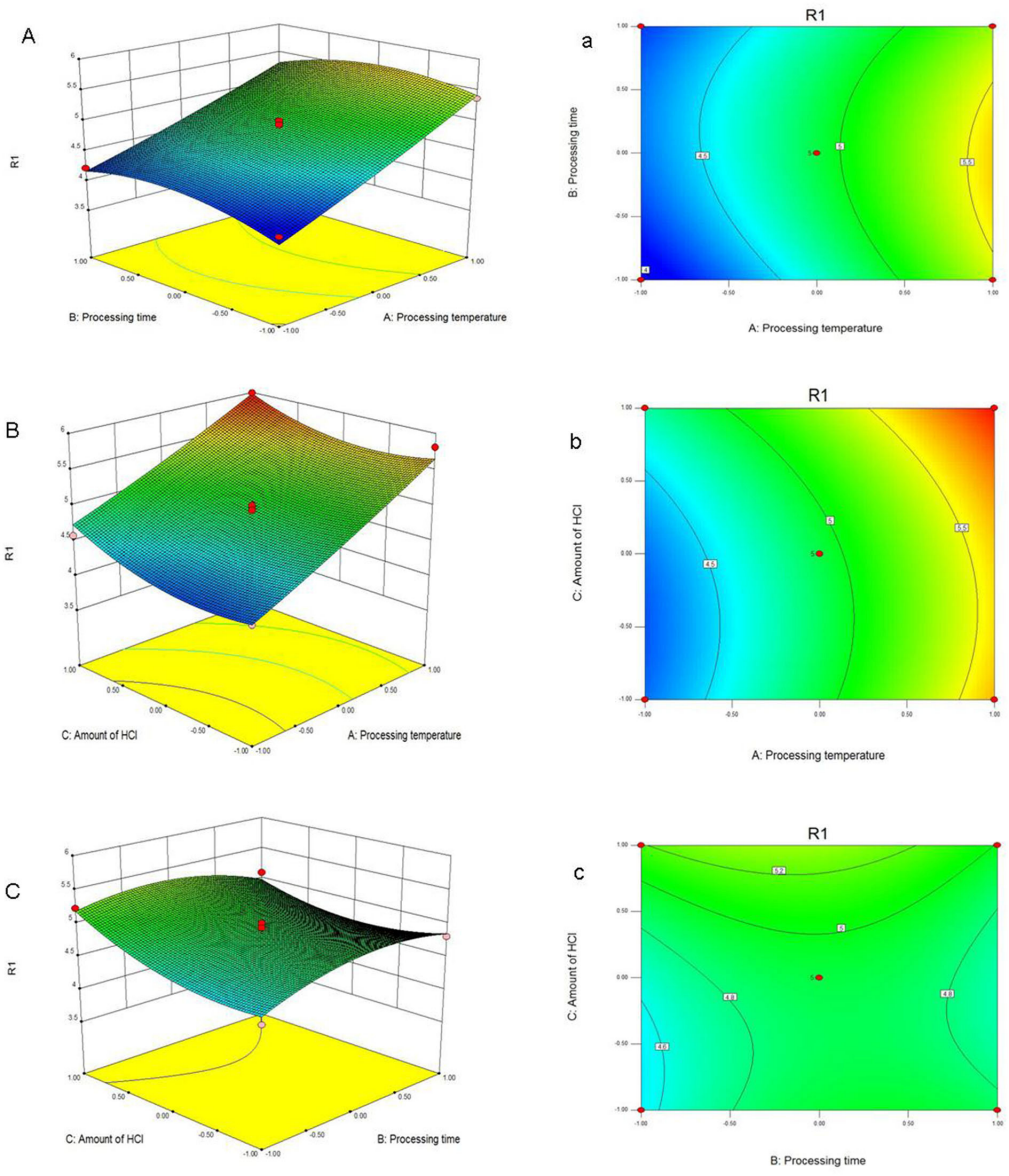
Response surface plots **(A–C)** and contour plots **(a–c)** showing the effects of processing temperature, processing time, amount of HCl and their mutual effects on the extraction yield of PFSV (R1).

**Figure 2:**
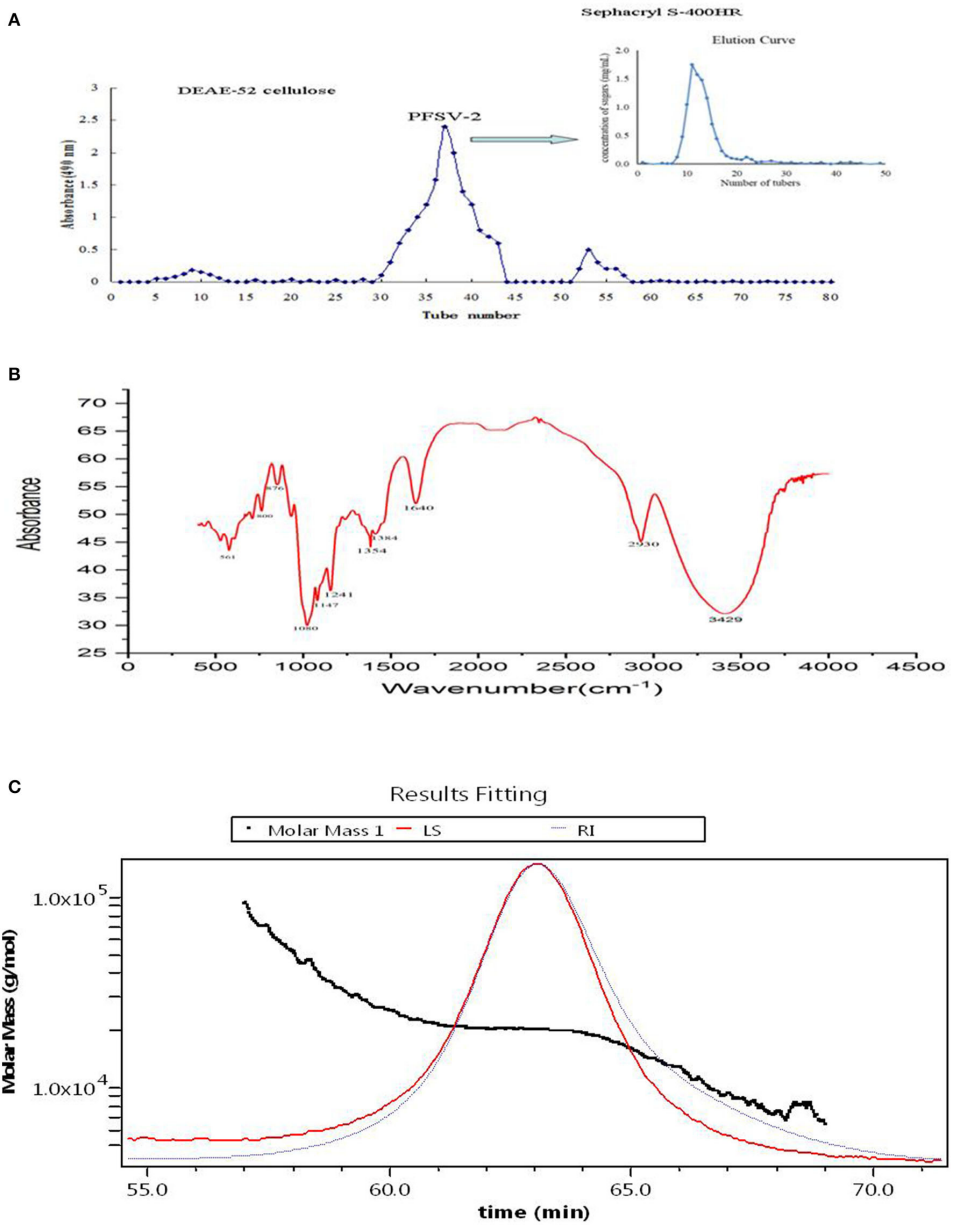
Elution curve of crude PFSV on DEAE-52 cellulose and sephacryl S-400HR chromatography column **(A)**; FT-IR spectrum of PFSV-2 **(B)**; and chromatograms of the molar mass distribution of PFSV-2 **(C)**.

According to this model, the linear terms of processing temperature and amount of HCl were the significant factors impacting the yield of PFSV, with the quadratic terms of processing time and amount of HCl indicating that these variables had the largest effects. The coefficients of the other terms were not significant. The 3D response surface (Figures 1A–C) and 2D contour plots (Figures 1a–c) provided a vivid portrayal of the effects of the variables as well as their interactions on the yield of polysaccharides. By analyzing the regression equation and the response surface contour plots, the optimal extraction conditions of PFSV were determined as follows: processing temperature, 80°C; processing time 0.81 h; amount of HCl, 1.5 ml. The maximum predicted extraction yield of PFSV was 6.02%, which corresponded well with the actual yield (5.94 ± 0.16%, *n* = 3). The above results indicated that the model designed in this study was valid.

The yields of PFSV, which could be achieved by the acid-ethanol pretreatment using the optimized procedure, were compared with those obtained by traditional extraction methods (HWE and UAE). The yields of polysaccharides obtained by HWE and UAE were 1.31 ± 0.15% and 2.04 ± 0.25%, respectively. Therefore, the PFSV yield obtained by acid-ethanol pretreatment in this study was 3.53-fold and 1.91-fold higher compared with conventional HWE and UAE, respectively (Supplementary Figure S1). To summarize, the yields of PFSV obtained by acid-ethanol pretreatment were much higher than those obtained using the other two traditional extraction methods.

### Purification of the crude PFSV and characterization of the purified polysaccharide

Using DEAE-52 cellulose column chromatography, PFSV was fractionated to obtain three fractions (Figure 2A). The second fraction was the main fraction, containing 89.8% of PFSV. This was further purified on a Sephacryl S-400HR column to obtain PFSV-2. *High-performance anion-exchange chromatography* analysis revealed that PFSV-2 was composed of fucose, rhamnose, galactose, glucose, xylose, and mannose in molar ratios of 13.50, 0.27, 32.59, 7.01, 0.85, and 15.82, respectively. Fucose, galactose, and mannose were the most abundant monosaccharides. The FT-IR spectra of PFSV-2 are shown in Figure 2B. The peak at 3,348 cm^−1^ (O–H groups) corresponded to the main characteristic polysaccharides peak (18). The absorption peaks at 2,930 and 1,417 cm^−1^ were assigned to the asymmetric stretching vibrations of C–H. The absorption at 1,640 cm^−1^ corresponded to the C=O stretching vibration. The broad peak around 1,404 cm^−1^ indicated the presence of uronic acid in PFSV-2. The absorptions at 1,078 and 876 cm^−1^ indicated that α- and β-configurations were both present. The skeletal modes of the pyranose rings were reflected in the bands in the 350–600 cm^−1^ range. The molecular weight parameters of PFSV-2 were determined by HPSEC-MALLS-RI. As shown in Figure 2C, the Mn, Mp, Mw, and Mz values of PFSV-2 were 17.497, 20.377, 19.256, and 21.193 KDa, respectively. The polydispersity indices (Mw/Mn and Mz/Mn) were 0.99 and 1.21, indicating that PFSV-2 was a homogeneous polysaccharide with a relatively narrow molecular weight distribution.

Methylation and GC-MS methods were used to determine the glycosidic bond types in PFSV-2. The sugar residues of PFSV-2 are summarized in Table 2 and their corresponding mass spectra are shown in Supplementary Figure S3. Nine derivatives were identified from the PFSV-2 methylation products. Among these monosaccharide residues, 6-linked Gal*p* accounted for the largest proportion of the total sugar residues, suggesting that the backbone of PFSV-2 may be composed of 6-linked Gal*p*.

**Table 2:** The PMAAs derived from PFSV-2.

**Retention time (min)**	**Molecular weight**	**Major mass fragments (m/z)**	**PMAAS**	**Molar ratios**	**Linkage**
6.66	293	59, 72, 102, 118, 131, 162, 175	1,5-di-O-acetyl-6-deoxy-2,3,4-tri-O-methyl fucitol	2.623	t-Fuc*p*
8.47	323	59, 71, 87, 102, 129, 145, 205	1,5-di-O-acetyl-2,3,4,6-tetra-O-methyl mannitol	15.061	t-Man*p*
9.54	323	59, 71, 87, 118, 145, 161, 205	1,5-di-O-acetyl-2,3,4,6-tetra-O-methyl galactitol	0.888	t-Gal*p*
9.97	321	59, 72, 89, 115, 131, 175, 190, 234	1,2,5-tri-O-acetyl-6-deoxy-3,4-di-O-methyl fucitol	11.808	2-Fuc*p*
11.95	351	57, 71, 88, 101, 129, 161, 190, 233	1,2,5-tri-O-acetyl-3,4,6-tri-O-methyl glucitol	1.571	2-Glc*p*
13.16	351	71, 87, 102, 129, 145, 162, 189, 205	1,5,6-tri-O-acetyl-2,3,4-tri-O-methyl mannitol	1.347	6-Man*p*
13.31	351	71, 87, 102, 118, 162, 189, 233	1,5,6-tri-O-acetyl-2,3,4-tri-O-methyl glucitol	1.631	6-Glc*p*
15.04	351	71, 87, 102, 118, 162, 189, 233	1,5,6-tri-O-acetyl-2,3,4-tri-O-methyl galactitol	47.375	6-Gal*p*
19.19	379	74, 87, 114, 130, 190, 234	1,2,5,6-tetra-O-acetyl-3,4-di-O-methyl mannitol	17.696	2,6-Man*p*

**Figure 3:**
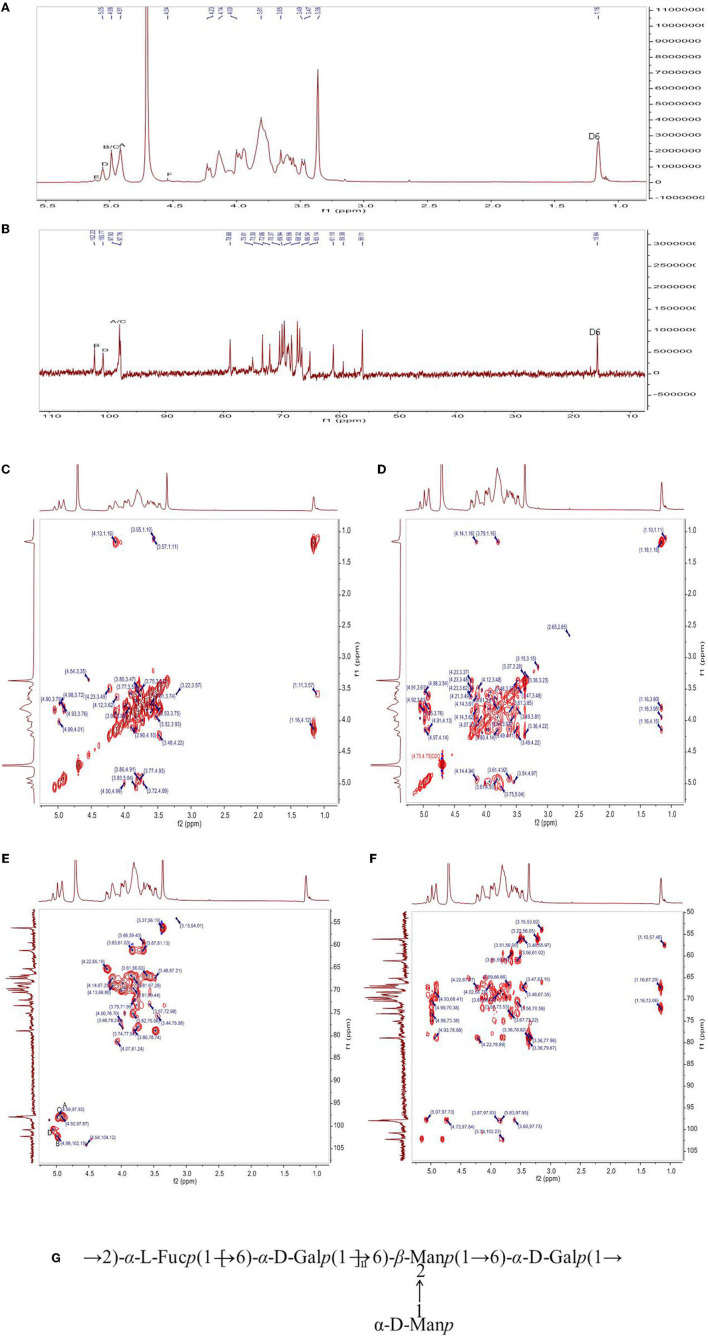
The ^1^H-NMR **(A)**, ^13^C **(B)** NMR, ^1^H-^1^H COSY **(C)**, NOESY **(D)**, HSQC **(E)**, and HMBC **(F)** spectra of PFSV-2. The predicted structure of PFSV-2 **(G)**.

The chemical structure of PFSV-2 was further determined using ^1^H, ^13^C, COSY, HSQC, and HMBC NMR spectroscopy (Figure 3). In the ^1^H spectrum (Figure 3A), the 3.2–5.3 ppm anomeric region was crowded. Six coupling signal peaks were identified from 4.4–5.4 ppm, indicating that there were at least six types of sugar residues in PFSV-2. The resonances between 3.3 and 4.1 ppm were assigned to the ring protons and highlighted the presence of the pyranose form (19).

The anomeric configuration of each residue in PFSV-2 was elucidated using the ^13^C NMR spectroscopy (Figure 3B). The chemical shifts of the anomeric carbons ranged from 95 to 110 ppm. Three strong signal peaks appeared at 102.23, 100.78, and 97.8 ppm, indicating that there was overlap between the anomeric carbon signals of the residues. The strong ^13^C signal at 56 ppm was assigned to the –O-CH_3_, which indicated its high content. It is more likely to link the → 6)-α-Gal*p*-(1 → at C-2, C-3 or C-4. In combination with the results of methylation analysis, HSQC, and ^13^C NMR and the residue classification, the anomeric carbon signals of the residues A, B, C and D (Figure 3C) were identified. Residues A, B, C and D were → 6)-α-Gal*p*-(1 → and → 2,6)-β-Man*p*-(1 → , t-α-Man*p*-(1 → , and → 2)-α-Fuc*p*-(1 → , respectively. The chemical shifts of these glycosidic linkages in PFSV-2 were assigned and are listed in Table 2.

Based on the chemical shifts of all the sugar residues and in combination with the HMBC results, the linkage sites and sequences of different sugar residues in PFSV-2 were analyzed. As suggested by the HMBC spectrum of PFSV-2 (Figure 3D), the correlation peaks at 4.91/69.07 ppm (A H-1/A C-6), 3.57/97.93 ppm (B H-6/A C-1), 3.81/102.23 ppm (A H-6/B C-1), 4.98/69.91 ppm (C H-1/B C-2), and 5.50/69.07 ppm (D H-1/A C-6) indicated that the O-1 of residue A was linked to C-6 of A, O-6 of residue B was linked to the C-1 of residue A, O-6 of residue A was linked to the C-1 of residue B, O-1 of residue C was linked to the C-2 of residue B, and O-1 of residue D was linked to the C-6 of residue A. Furthermore, in the NOESY results (Figure 3E), cross-peaks appeared at (A H1, A H6, D H2), (B H1 and A H6) and (D H1 and A H6). In combination with the previous results, this enabled the verification of the linkage sites and sequences of different sugar residues in PFSV-2 and the primary structure of PFSV-2 to be deduced, as shown in Figure 3F. The backbone of PFSV-2 was composed of → 6)-α-Gal*p*-(1 → and → 2,6)-β-Man*p*-(1 → and → 2)-α-Fuc*p*-(1 → and branched of t-α-Man*p*-(1 → at position 2 of residue B.

### Lipid lowering activity

The zebrafish hyperlipidemia model is commonly used for the assessment of hypolipidemic drugs, as zebrafish larvae possess important organs for energy balance and metabolism, such as lipid storage in white adipocytes (20). ORO staining can be used to visualize neutral lipid localization in whole zebrafish (21). As shown in Supplementary Figure S3, minimal ORO staining was found in the control group (without feeding egg yolk) throughout the culture period. In contrast, in the egg yolk group, the lipid was abundant in the gut, yolk sac and blood vessels. As expected, compared to the egg yolk control, PFSV-2 at concentrations of 0.5 and 1.0 mg·ml^−1^ significantly decreased the lipid content. For the positive group, simvastatin also remarkably lowered the lipid level compared to the egg yolk group. These results manifested that PFSV-2 is a potent hypolipidemic agent.

**Figure 4:**
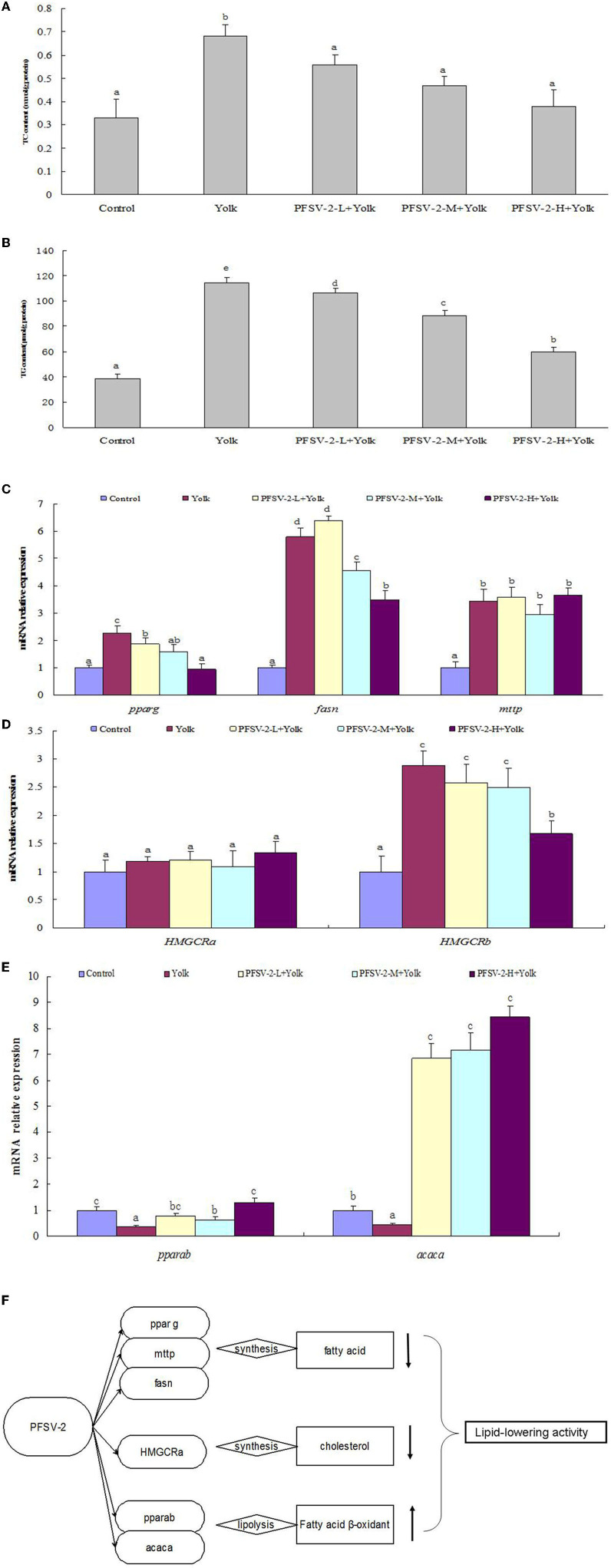
Total cholesterol [TC, **(A)**] and triglyceride [TG, **(B)**] levels of larval zebrafish in each group (*n* = 3); mRNA expression of *pparg, fasn*, and *mtp* on zebrafish larvae **(C)**; mRNA expression of *pparab* and *acaca* on zebrafish larvae **(D)**, mRNA expression of *HMGCRa* and *HMGCRb* on zebrafish larvae **(E)**, and molecular mechanisms of lipid metabolism modulation by PFSV-2 **(F)**. Bar indicates means ± SD. Bars without the same superscripts (a–e) denote significant difference (*p* < 0.05).

As shown in Figures 4A, B, compared with the control group, the TG and TC content of the egg yolk group was significantly increased (*p* < 0.05). Following treatment with PFSV-2 for 3 days, the content of TG and TC was significantly reduced as compared to the egg yolk group. Interestingly, the lipid-modulating effect of PFSV-2 showed concentration dependence. This result confirmed the results of ORO staining; that is, the decrease in lipid levels following treatment by PFSV-2.

### Effects of PFSV-2 on the expression of lipid metabolism genes in zebrafish

Abnormal lipid accumulation and deposition can be induced by an imbalance between lipogenesis and lipolysis. Therefore, the hypolipidemic activity of PFSV-2 depends on the facilitation of lipolysis or inhibition of lipogenesis. For lipogenic genes, the effects of PFSV-2 on the expression levels of peroxisome proliferator-activated receptor gamma (*pparg*), fatty acid synthase (*fasn*) and microsomal triglyceride transfer protein (*mtp*) were determined. As shown in Figure 4C, the expressions of the fatty acid synthesis related genes *pparg* and *fasn* were upregulated significantly in egg yolk group compared with the control group. Importantly, the expression of these genes was significantly downregulated by PFSV-2 in yolk-treated zebrafish larvae. However, there was no significant difference in *mtp* gene expression between the egg yolk group and PFSV-2 treatment groups.

To investigate the effects of PFSV-2 on cholesterol biosynthesis genes, the mRNA levels of *HMGCRa* and *HMGCRb* (the rate-limiting enzymes of cholesterol synthesis in zebrafish) were investigated (Figure 4D). The results indicated that *HMGCRb* expression were notably elevated in the egg yolk group but remarkably decreased in the PFSV-2 treatment group. However, the *HMGCRa* gene expression was not significantly different in any of the tested groups. Thus, PFSV-2 may exert its hypocholesterolemic effect by decreasing the levels of *HMGCRb*.

To explore the effects of PFSV-2 on lipolysis, mainly beta-oxidation, the mRNA expression levels of peroxisome proliferator-activated receptor alpha b (*pparab*) and acetyl-CoA carboxylase alpha (*acaca*) were determined (Figure 4E). The expressions of *pparab* and *acaca* were remarkably decreased in the egg yolk group but significantly upregulated by PFSV-2.

Various methods have been reported for the extraction of polysaccharide from *S. vaninii*. Chan et al. (22) extracted polysaccharides from *S. vaninii* using hot water, microwave-assisted extraction, maceration, and ultrasonic-assisted extraction, achieving yields of 2.17%, 2.25%, 2.02%, and 2.11%, respectively. In the present study, the yields of PFSV-2 extracted by HWE and UAE were 1.31 ± 0.15% and 2.04 ± 0.25%, respectively. These results were similar to those reported above. However, the PFSV-2 yield obtained by acidic ethanol pretreatment reached 5.94 ± 0.16%. This result confirmed that the acid-ethanol pretreatment plays an important role when aiming to high polysaccharide yield. Disruption not only enabled solvent to penetrate the cell wall but also enhanced polysaccharide dissolution.

In the present study, the zebrafish was used to investigate the lipid-lowering activity of PFSV-2. Zebrafish on a high-calorie diet showed hyperlipidemia, as verified by ORO staining and high TG and TC levels. PFSV-2 treated larvae showed smaller area of ORO staining and lower TG and TC content. These results indicated that PFSV-2 to be a potent lipid-lowering agent.

To investigate the underlying mechanism of the hypolipidemic properties of PFSV-2, seven lipid metabolism-related genes expression of *pparg, fasn, mtp, HMGGRa, HMGGRb, pparab* and *acaca* were investigated by RT-PCR. As one of the key factors driving adipogenesis, *pparg* can promote lipid storage in white adipose tissue as well as preadipocyte differentiation to mature adipocytes (23). The activation of *pparg* ultimately promotes the uptake, synthesis, esterification and storage of fatty acid in newly formed adipose cells (24). In the present study, the expression of *pparg* was significantly increased in the egg yolk group, which was in disagreement with the results of Wang et al. (25), who found that there were no changes to *pparg* in the egg yolk group. The low levels of *pparg* in PFSV-2 treated zebrafish larvae showed that PFSV-2 antagonized lipid storage in adipose tissue and impeded preadipocytes differentiation to mature adipocytes.

The gene *fasn* can facilitate the synthesis of long-chain fatty acids. It is minimally expressed in normal cells and highly expressed in various pathological conditions (26). The findings of the present study were consistent with previous reports (27), that is, the expression of *fasn* was significantly increased in the egg yolk group and suppressed by PFSV-2, compared with the control. These results suggested that the curative properties of PFSV-2 against hyperlipidemia are partly caused by suppression of the lipogenesis gene *fasn*.

*HMGCRb* is the key enzyme involved in lipid metabolism regulation and cholesterol biosynthesis in zebrafish larvae and an essential involved gene in the synthesis of TC (28). *HMGCRb* is the target of a cholesterol-lowering drugs-statins (29). In the present study, PFSV-2 significantly downregulated the elevated mRNA levels of *HMGCRb* in zebrafish larvae. Thus, PFSV-2 may exert its hypocholesterolemic effect by decreasing the level of *HMGCRb*.

The *acaca* gene can regulate β-oxidation of mitochondrial fatty acid and affect the expression of lipid metabolism (30). Additionally, the activation of *pparab* can decrease lipid levels *via* the enhancement of fatty acid β-oxidation in the liver (31). In the present study, the expression of *pparab* and *acaca* was remarkably decreased in the egg yolk group but significantly upregulated by PFSV-2. These results indicated that PFSV-2 may promote β-oxidation of fatty acid through upregulating the expression of *pparab* and *acaca*.

The hypolipidemic effects of polysaccharides have been demonstrated in many studies. Polysaccharides from cassia seeds can bind to bile acids, thereby decreasing the absorption of dietary cholesterol (32). Polysaccharides from *Auricularia auricular* displayed a hypolipidemic effect against high-fat emulsion-induced hyperlipidemia (33). Polysaccharides derived from *Grifola frondose* and *Catathelasma ventricosum* showed hypolipidemic activity in streptozotocin-induced diabetic mice (34, 35). And polysaccharide from *Agaricus bisporus* can impede the lipid accumulation in zebrafish models mainly through the regulation of adipogenesis (36).

Hypolipidemic activity of polysaccharide mainly depends on their molecular weight, sulfate content, sulfate position, type of linkage and molecular geometry (37). Laminarin sulfates with highest sulfate content have shown maximum hypolipidemic activity (38). Polysaccharides from *Gastrodia elata* Blume, which consisted mainly of glucose, exhibited a marked hypolipidemic effect in rats (39). Molecular weight has been demonstrated to affect the bile acid-binding ability of polysaccharides from *Plantago asiatica* seeds (40). The high choate-binding ability of polysaccharide was reported due to its lower molecular weight and more exposed hydroxyl groups (34). Zeng et al. (41) reported that the hypolipidemic effect of polysaccharide from *Fortunella margarita* (Lour.) Swingle was affected by a combination of multiple structural factors. It was also reported that the potent lipid-lowering potential of the polysaccharide partly by alleviating oxidative stress (42). The inherent mechanism underlying the hypolipidemic activity of PFSV-2 will be investigated in future research.

## Conclusion

Polysaccharides were extracted from *S. vaninii* by the acid-ethanol pretreatment. RSM and BBD were used to determine the optimal processing conditions as follows: processing temperature, 80°C, processing time, 0.81 h and amount of HCl, 1.5 ml. Under these conditions, the extraction yield of crude PFSV reached 5.94 ± 0.16%. The purified polysaccharide was successfully obtained by multiple column chromatography and its physicochemical and structural properties were investigated. Bioactivity assays indicated that PFSV-2 exhibited effective lipid-lowering activity with significantly decreased lipid accumulation and levels of TC and TG in zebrafish larvae. Furthermore, PFSV-2 downregulated the lipogenic genes (*pparg* and *fasn*) and cholesterol biosynthesis gene (*HMGCRb*) and upregulated the lipolysis genes (*pparab* and *acaca*) in zebrafish larvae (Figure 4F). To conclude, the acid-ethanol pretreatment was a superior method for the extraction of polysaccharide from *S. vaninii* and PFSV-2 exhibits the hypolipidemic properties *via* lipid metabolism-related pathways.

## Data availability statement

The original contributions presented in the study are included in the article/Supplementary material, further inquiries can be directed to the corresponding authors.

## Ethics statement

The animal study was reviewed and approved by the Guide for the Care and Use of Laboratory Animals. Written informed consent was obtained from the owners for the participation of their animals in this study.

## Author contributions

Z-fZ: funding acquisition, review, and editing. T-tS: data curation. J-fC: visualization. G-yL: project administration and investigation. All authors contributed to the article and approved the submitted version.
