# Supplementary material for: Recovery of a hypolipidemic polysaccharide from artificially cultivated Sanghuangporus vaninii with an effective method
Source: Front Nutr. 2023 Jan 13;9:1095556. doi: 10.3389/fnut.2022.1095556 (PMC9880258; doi:10.3389/fnut.2022.1095556)
Supplement: Supplementary file 1 [file Table_1.DOC]

**Table S1** Primers used for real-time quantitative PCR (qRT-PCR)

| Gene name | Primer sequence | Product sizes | Note |
| --- | --- | --- | --- |
| *β-actin* | ACGAACGACCAACCTAAACTCT | 114 | qRT-PCR |
|  | TTAGACAACTACCTCCCTTTGC |  |  |
| *HMGCRa* | CTAGTCGCCAGCACAAACAG | 135 | qRT-PCR |
|  | CTTGACTTCAGCCGCTTGAC |  |  |
| *HMGCRb* | AATGGCAATACTGGGACCCA | 106 | qRT-PCR |
|  | ACAGCACATGATCTCCAGCT |  |  |
| *pparab* | CGTCGTCAGGTGTTTACGGT | 250 | qRT-PCR |
|  | AGGCACTTCTGGAATCGACA |  |  |
| *pparg* | GGCGCTTCAGTGTTCAGAAA | 147 | qRT-PCR |
|  | CAGCTCCTCCAGTTCCAGAG |  |  |
| *acaca* | GATGGTGGCTGACGTTAACC | 113 | qRT-PCR |
|  | AATCTCCCCATGGCTGCAT |  |  |
| *fasn* | CCATTCTGGCTGCTTACTGG | 102 | qRT-PCR |
|  | GAGCTTTGCACTCTTCCCAC |  |  |
| *mtp* | GCGCAAATCCAGCAAGTCT | 102 | qRT-PCR |
|  | GATGGAGTCTGGGCTGATGT |  |  |
