# Supplementary material for: Recovery of a hypolipidemic polysaccharide from artificially cultivated Sanghuangporus vaninii with an effective method
Source: Front Nutr. 2023 Jan 13;9:1095556. doi: 10.3389/fnut.2022.1095556 (PMC9880258; doi:10.3389/fnut.2022.1095556)
Supplement: Supplementary file 2 [file Data_Sheet_1.doc]

A

**Figure S1** Comparison of the extraction rates of polysaccharides of the three methods.

AEP: acid ethanol pretreatment; HWE: hot water extraction; UAE: ultrasonic assisted extraction

**Supplementary Fig 1.** Scanning electron micrographs (200×) of *Sanghuangporus vaninii materials* before (A) and after (B)the extraction process; Comparison of the extraction rates of polysaccharides of the three methods.

AEP: acid ethanol pretreatment; HWE: hot water extraction; UAE: ultrasonic assisted extraction
