# Supplementary material for: Recovery of a hypolipidemic polysaccharide from artificially cultivated Sanghuangporus vaninii with an effective method
Source: Front Nutr. 2023 Jan 13;9:1095556. doi: 10.3389/fnut.2022.1095556 (PMC9880258; doi:10.3389/fnut.2022.1095556)
Supplement: Supplementary file 3 [file Data_Sheet_2.doc]

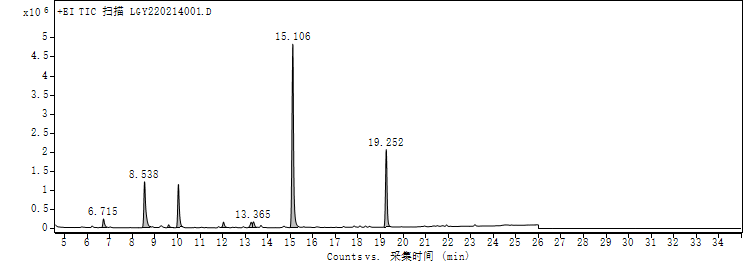


az


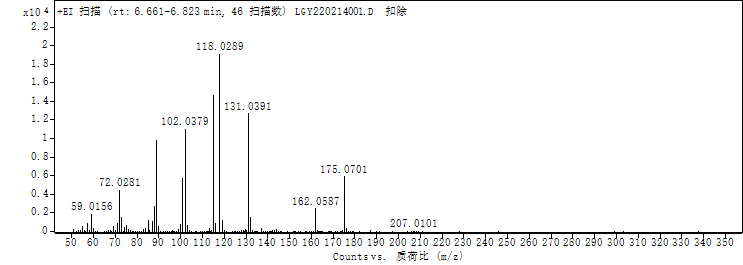


bz


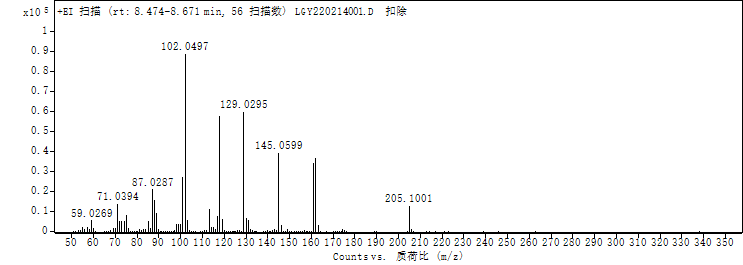


cz


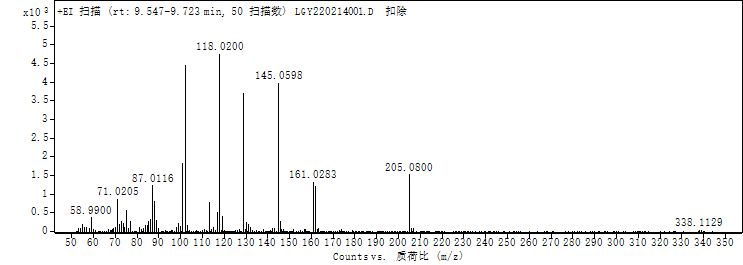


d


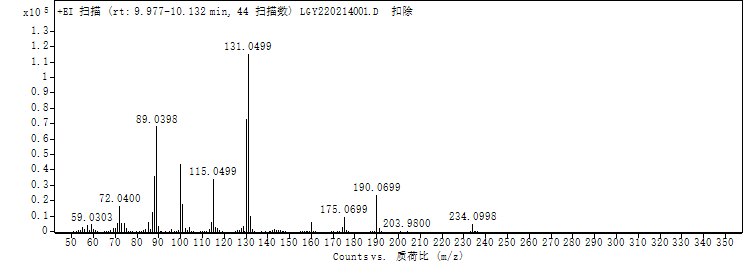


ez


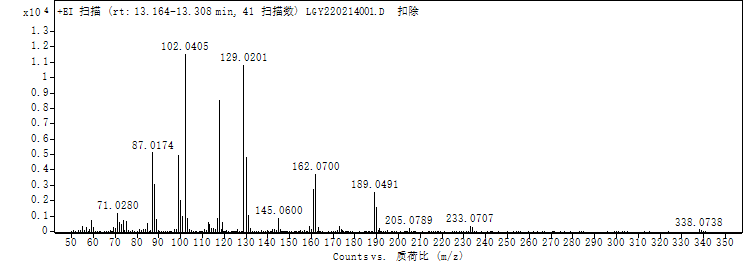

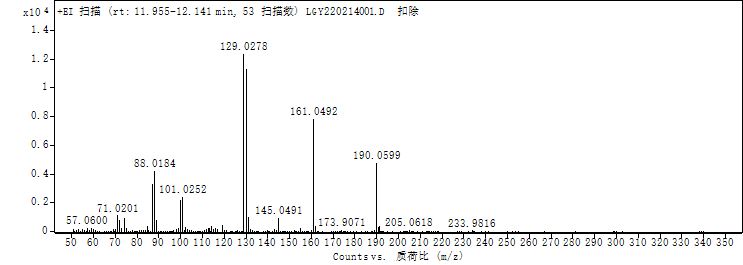


fz

gz


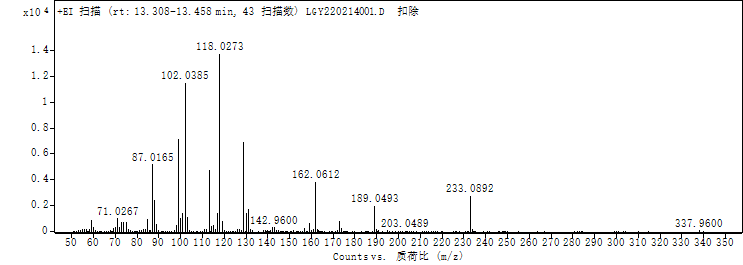


hz


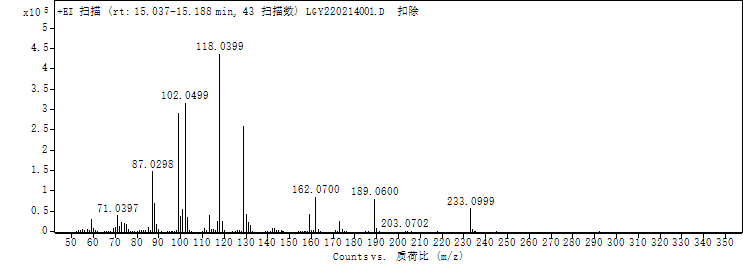


iz


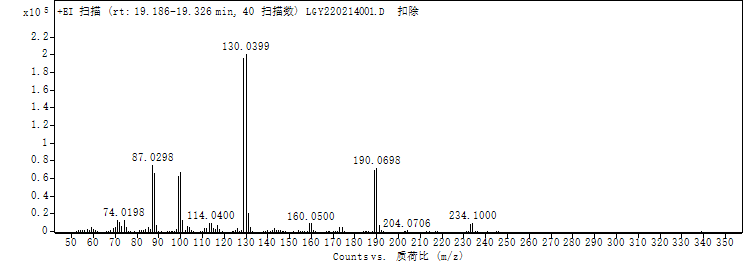


jz

**Figure S2** The EI-MS spectrum of PFSV. Total ion chromatogram of PFSV derivatives by GC-MS(a); The mass spectra of 1,5-di-O-acetyl-6-deoxy-2,3,4-tri-O-methyl fucitol at 6.66 min(b); The mass spectra of 1,5-di-O-acetyl-2,3,4,6-tetra-O-methyl mannitol at 8.47 min (c); The mass spectra of 1,5-di-O-acetyl-2,3,4,6-tetra-O-methyl galactitol at 9.54 min(d); The mass spectra of1,2,5-tri-O-acetyl-6-deoxy-3,4-di-O-methyl fucitol at 9.97 min (e); The mass spectra of 1,2,5-tri-O-acetyl-3,4,6-tri-O-methyl glucitol at 11.95 min (f); The mass spectra of 1,5,6-tri-O-acetyl-2,3,4-tri-O-methyl mannitol at 13.16 min (g) ; The mass spectra of 1,5,6-tri-O-acetyl-2,3,4-tri-O-methyl glucitol at 13.31 min (h); The mass spectra of 1,5,6-tri-O-acetyl-2,3,4-tri-O-methyl galactitol at 15.04 min (i); The mass spectra of 1,2,5,6-tetra-O-acetyl-3,4-di-O-methyl mannitol at 19.19 min (j).
