# Supplementary material for: Recovery of a hypolipidemic polysaccharide from artificially cultivated Sanghuangporus vaninii with an effective method
Source: Front Nutr. 2023 Jan 13;9:1095556. doi: 10.3389/fnut.2022.1095556 (PMC9880258; doi:10.3389/fnut.2022.1095556)
Supplement: Supplementary file 4 [file Data_Sheet_3.doc]

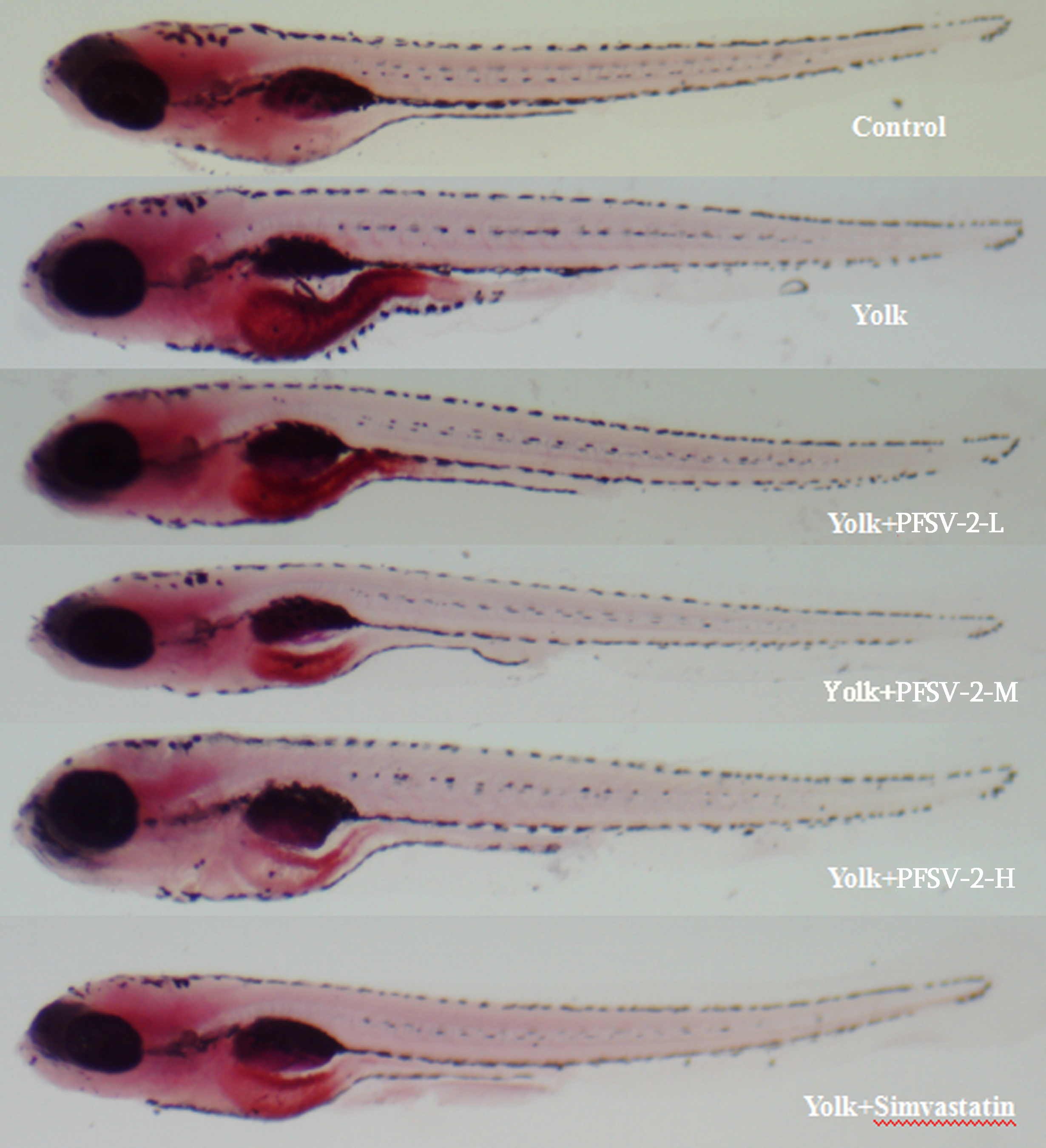


**Figure S3.** Lipid-lowering effect of PFSV-2 in a zebrafish hyperlipidemia model. Light microscope images of ORO-stained zebrafish larvae were captured at 30 × magnification.
